# A Risk Assessment Model for Type 2 Diabetes in Chinese

**DOI:** 10.1371/journal.pone.0104046

**Published:** 2014-08-07

**Authors:** Senlin Luo, Longfei Han, Ping Zeng, Feng Chen, Limin Pan, Shu Wang, Tiemei Zhang

**Affiliations:** 1 Information System and Security & Countermeasures Experimental Center, Beijing Institute of Technology, Beijing, P. R. China; 2 Beijing Institute of Geriatrics, Beijing Hospital, Ministry of Health, Beijing, P. R. China; 3 College of Public Health, Nanjing Medical University, Nanjing, Jiangsu, P. R. China; Tor Vergata University of Rome, Italy

## Abstract

**Aims:**

To develop a risk assessment model for persons at risk from type 2 diabetes in Chinese.

**Materials and Methods:**

The model was generated from the cross-sectional data of 16246 persons aged from 20 years old and over. C4.5 algorithm and multivariate logistic regression were used for variable selection. Relative risk value combined with expert decision constructed a comprehensive risk assessment for evaluating the individual risk category. The validity of the model was tested by cross validation and a survey performed six years later with some participants.

**Results:**

Nine variables were selected as risk variables. A mathematical model was established to calculate the average probability of diabetes in each cluster's group divided by sex and age. A series of criteria combined with relative RR value (2.2) and level of risk variables stratified individuals into four risk groups (non, low, medium and high risk). The overall accuracy reached 90.99% evaluated by cross-validation inside the model population. The incidence of diabetes for each risk group increased from 1.5 (non-risk group) to 28.2(high-risk group) per one thousand persons per year with six years follow-up.

**Discussion:**

The model could determine the individual risk for type 2 diabetes by four risk degrees. This model could be used as a technique tool not only to support screening persons at different risk, but also to evaluate the result of the intervention.

## Introduction

Type 2 diabetes is a worldwide public health problem resulting from both lifestyle and genetic factors. Although the pathogenesis of diabetes is unclear, fortunately, type 2 diabetes can be prevented by available lifestyle intervention [1].

High risk intervention is one of the main strategies in non-communicable diseases prevention, which is also suitable for type 2 diabetes. One of problems is how to find and determine those at high risk within a population. Based on epidemiological research work, the risk factors associated with the onset of diabetes, especially type 2 diabetes, were clear. A set of assessment methodologies, from the simple checking list to several risk score models, was developed in recent years, such as the Finnish Risk Score, Danish Diabetes Risk Score [2], ADA [3], Cambridge Risk Score [4], NHANESIII [5], DRC [6], Thailand Risk Score [7], Spanish Diabetes Risk Score [8] and so on [9], [10]. All these tools are helpful in the assessment of high risk and of persons with diabetes all over the world but only a few are developed in China [11], [12].Considering the large number of individuals with pre-diabetes or at high-risk of diabetes in China [13], a tool, suitable for Chinese and with a high efficiency for dealing with the huge clinical data simultaneously in a precise way, is urgently needed.

The aim of this work was to develop a risk assessment model for type 2 diabetes in China, which was an available and affordable computerized model with common variables. The model could be used to assess different levels of risk for type 2 diabetes both at the individual's level and at the population's level, and therefore, to support the prevention of diabetes.

## Materials and Methods

### Subjects

Form Mar to Nov. 2001, a cross-sectional baseline survey was drawn from 27 research institutes in Beijing, China. Most of the research institutes are located in the urban districts of Xicheng, Haidian and Shijingshan. A total of 16246 subjects aged 20 and older were enrolled when they participated in their annual health examination through a questionnaire on health behavior and clinical measurements. Almost all persons are researchers or graduated students (younger than 30 years old). These persons were highly educated and in a sedentary working pattern. Health behavior included age, sex, prior history of diabetes and parental or sibling history of diabetes (PSH), clinical examination included the measurements of height, weight, waist circumference, systolic blood pressure (SBP), diastolic blood pressure (DBP), cholesterol (CHOL), triglyceride (TG), high-density lipoprotein (HDL), low-density lipoprotein (LDL), and fasting plasma glucose (GLU). Body mass index (BMI) was calculated dividing the weight (kg) by the height squared (m2). The investigations were executed by trained medical staff from these institutes. All the blood test were conducted by one clinical biochemistry department in an evaluated hospital. The baseline survey recruited 16246 persons. Among them were 15237 persons who were free of a history of diabetes and fasting glucose levels <7.0 mmol/L, 1009 persons who have a history of diabetes or fasting glucose levels >7.0. Six years later, in 2007, during the same period as in 2001, the follow-up survey was carried out in four institutes of the above 27 institutes when about 2288 persons without diabetes in 2001 completed the follow-up investigation.

### Ethics Statement

The research was approved by the 12th Five-year National Science and Technology Supporting Project and National Natural Science Foundation of China, and all participants gave written consent to participation in the study. The individual in this manuscript has given written informed consent to publish these case details.

### Model Design and Methods

**Figure 1:**
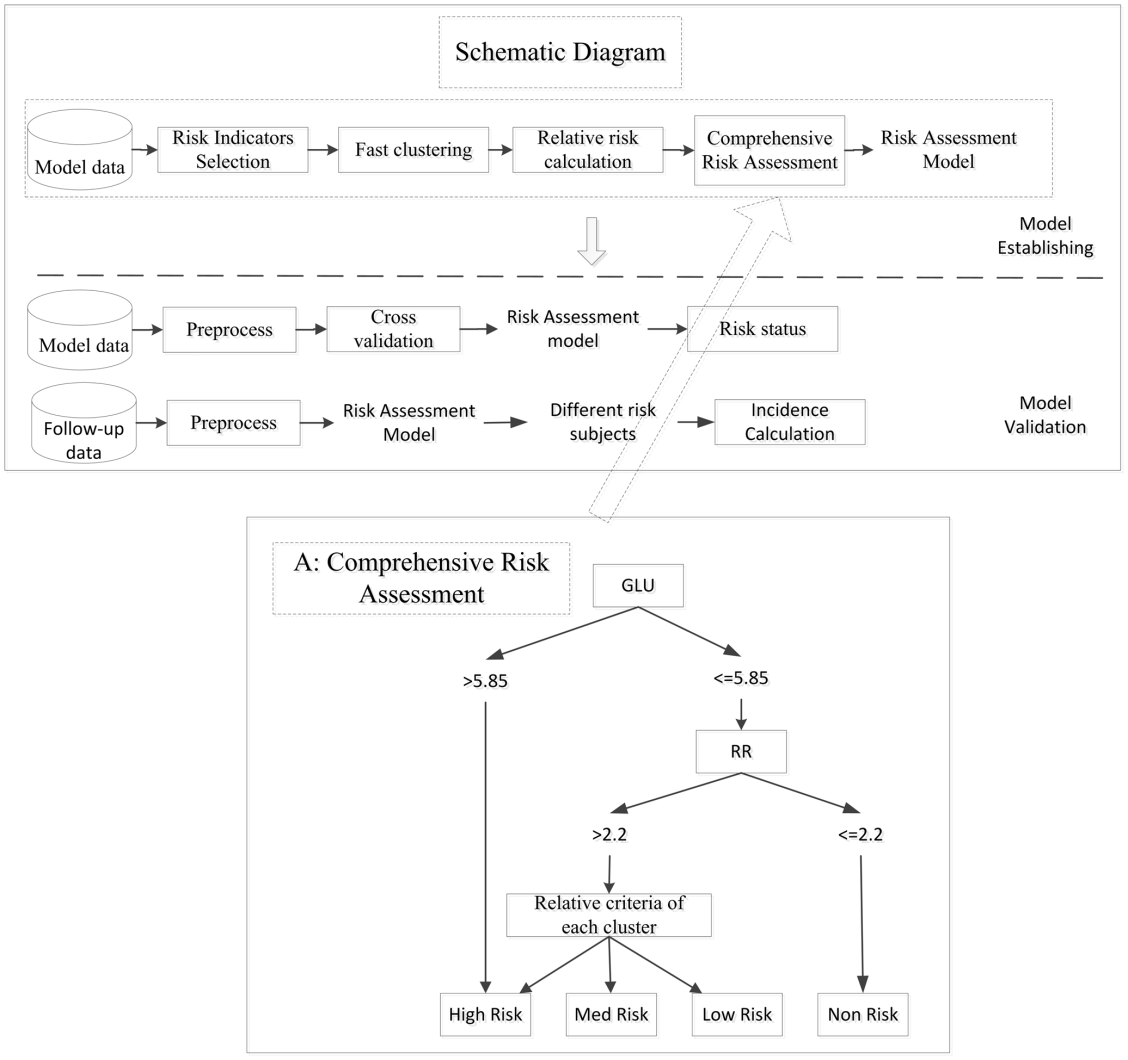
The schematic diagram of the Risk assessment Model and Comprehensive Risk Assessment Procedure.

Data mining and statistical methods combined with expert decisions were used to accomplish variable selection and analyze the impact of clinical variables on the risk of type 2 diabetes. The schematic diagram is shown in Figure 1.

### 1. Variable Selection

The variable selection was firstly filtered based on the preprocessing of the variables. The variables, which had large vacant data, were deleted. Then, the remaining variables were accomplished using C4.5 decision tree [14] and multivariate logistic regression. The same variables chosen both by C4.5 and logistic regression were regarded as the final risk variables.

The total number of questionnaire and clinical variables was 96. After variables filtering, 57 remained. The variables list was shown in Table S7 in File S1. Then, all the 16246 persons were divided into five sub-cohorts: (1) all persons; (2) all the males; (3) all the females; (4) all the persons under 50 year-old; (5) all the persons above 50 year-old. Therefore there were five cohorts as the training sets finally. C4.5 algorithm was applied to build the decision tree for each sub-cohort, the diabetic label was conducted as an identical class label. The C4.5 algorithm [15] was executed for each candidate training set by Weka [16] (Weka v.3-6-10, 2013, University of WAIKATO, Hamilton, New Zealand). The decision trees were obtained by 10-fold cross validation without pruning. The variables, whose frequencies of occurrence were higher than five of the first eight levels of the decision trees, would be the most relevant variables.

Meanwhile, we put the same 57 variables into multivariate logistic regression. The variables which were statistically significant (P<0.05) would be selected as the most relevant variables. The multivariate logistic regression was executed by Weka. Finally, age, diastolic blood pressure (DBP), high-density lipoprotein (HDL), waist, sex, cholesterol (CHOL), parental or sibling history, body mass index (BMI), and triglyceride (TG) were both selected by C4.5 and logistic regression simultaneously. The C4.5 and LR results of variable selection are shown in Table S3 and Table S4 in File S1.

Furthermore, from the results of the C4.5 algorithm, the value around 5.85 mmol/L of glycemia appeared six times in the first eight layers and four times in the first two layers. So the value 5.85 mmol/L was chosen as the cut-off point of glycemia for persons at high risk. Concretely, if the level of an individual's glycemia was higher than 5.85 mmol/L, the individuals were considered as a person at high risk.

### 2. Fast Clustering

It was clear that all of the selected variables were not specific to diabetes. However, any single variable could not be used to identify the risk of diabetes. To extract the features of persons at risk, the fast clustering was used to divide the population into different clusters by all selected variables, which could lead to a greater similarity between persons within the same cluster and a greater diversity between persons in different clusters.

First, the 16246 data were standardized. Then, the standardized data were put into the fast clustering analysis (fastclus) [17]. Fastclus was performed to estimate the different characteristics of the data based on nine variables which had been selected as risk variables.

Two steps were applied to determine the most appropriate number of clusters: (1) Several runs of the fastclus procedure were executed by setting different cluster numbers from 2 to 7; (2) Square of R (R^2^) and cubic clustering criterion (CCC) [18] were calculated in each run. Generally, a smaller number of clusters enhances the ease of interpretation, and a higher increment of R^2^ and a higher CCC indicated a better separation of clusters. So the smaller cluster number was chosen by CCC ≥10, and the most significant increments of R^2^. The results of the preselected number of clusters are shown in Table S1 in File S1.

According to the results of R^2^ and CCC, the optimal cluster number was 3. The fastclus procedure was conducted by R [19] (R v3.0.2, R Foundation for Statistical Computing, Wirtschaftsuniversität Wien), and three cohorts were finally obtained.

### 3. Relative Risk Calculation

After the fastclus procedure, the model population was divided into three groups with relative significant similarities in each group. The characteristics of the persons in the three clusters are shown in Table S5 in File S1. The relative risk for individuals in each group was calculated by the following two steps.

First, the probability of diabetes for each person in each cluster was calculated. Second, relative risk was a ratio calculated by individual probability divided by average probability of the onset of diabetes, which was computed according to age and gender in his or her corresponding cluster.

#### 3.1 Logistic Regression

Logistic regression was needed to calculate the probability of the onset of diabetes both for individuals and for each cluster primarily.

For each cluster obtained by fast clustering, 16246 data from the different clusters were put into the multivariate logistic model. Then the goodness of fit for the logistic regression models was evaluated by the Hosmer and Lemeshow test [20]. Generally, a larger P value represented a better match. The Beta coefficient of each independent variable could be derived from the multivariate logistic regression for each cluster. The logistic regressions were built by R. The result of the Hosmer and Lemeshow test was shown in Table S2 in File S1.

#### 3.2 Average Probability of the Onset of Diabetes for Each Cluster

The 15237 subjects without diabetes were divided into 24 groups in each cluster by age (5 years a group between 20 to >75 years old) and gender. The means of variables for each group were put into the logistic regression of each matching cluster to calculate the average probability of the onset of diabetes in each group.

#### 3.3 Individual Relative Risk Calculation

The individual probability (P_k_) of the onset of diabetes was calculated one by one for 15237 subjects. The average probabilities (P_0_) of diabetes of the 24 groups in each cluster were also calculated by the mean value of variables of each group. The individual relative risk (RR) was calculated dividing individual probability P_k_ by average probability P_0_ of the age-gender matching group. The formula of RR is shown as below:
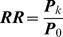



The ratio of probability between any subject and the age-gender matching group (RR) indicated the individual relative degree of the risk of diabetes.

A cut-off point of RR, to distinguish the individual with a risk of diabetes, was chosen by receiver-operating characteristic (ROC) curve [21]. Commonly, the optimal cut-off point was identified as the coordinate closest to the y intercept (0, 1) of the ROC curve. The ROC curve was executed by R, and the curve of all the subjects is shown in Figure S1 in File S1.

The area under the curve (AUC) of the ROC curve was 0.808. According to the criteria of choosing the optimal RR, the cut-off point of the RR value is 2.2, the sensitivity was 0.794, and specificity was 0.679.

### 4. Criteria of Comprehensive Risk Assessment

A criterion of comprehensive risk assessment (CCRA) was the final process in the construction of the risk assessment model. The CCRA executed in three steps (Figure 1). and started from (1) absolute criteria: whether the glycemia level of an individual was higher than the value 5.85 mmol/L or not; (2) relative risk assessment: whether the RR of an individual was higher than 2.2 or not; (3) relative criteria of each cluster: For the individuals whose RR was higher than 2.2, a relative criteria of each cluster was used to judge degree of the risk by selected risk variables combined with characteristics of the cluster and clinical knowledge. For example, in first cluster, if an individual satisfied all three of the following criteria (degree = 3): (1) BMI >26.8; (2) CHOL >5.18 mmol/L; (3) TG >1.7 mmol/L, this individual was assessed as being at high risk. If an individual satisfied any two of the three criteria (degree = 2), they were assessed as medium-risk. If they satisfied any one of the three criteria (degree = 1), the individuals were assessed as low-risk.

The pseudocode of the first cluster's main steps for risk assessment is shown below, other clusters were processed in a similar way to the first cluster but have different criteria. For the individuals whose RR was lower than 2.2, they would be identified as the non-risk individuals. The risk assessment model finally divided the model population into four risk categories: high-risk, medium-risk, low-risk and non-risk of diabetes.
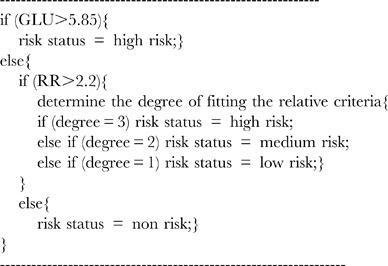



All the procedures of fastclus, relative risk calculation and the risk assessment were accomplished by C++ in computer, and the model was free of charge for the public. The website of the Risk Assessment Model for Type 2 Diabetes is http://www.isclab.org/rsd/RSDAssess.php.

### 5. Model Validation

The model validation was conducted in two steps: jackknife validation and follow-up validation.

In the jackknife validation, all 16246 subjects were classified into three categories manually: non-risk, different-risk (low-risk, medium-risk and high-risk) and subjects with diabetes. Then, the jackknife method was applied to proceed with the cross-validation. The jackknife method [22] randomly split 16245 data into one set to fit the model, and left the one last data to test the assessment accuracy. For a leave-one-out estimate, all 16246 data were used to get an overall estimate of the prediction accuracy based on the risk assessment model. Through these 16246 evaluations, the overall accuracy of these categories were calculated by the following formula. The jackknife was executed by R.
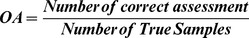



In the follow-up validation, a longitudinal data was used to validate the risk assessment model. The incidence of diabetes was calculated from a total of 2288 subjects without diabetes from the original model population in a follow-up six years later, in 2007. 62 persons developed diabetes during six-year follow-up. The incidence of diabetes from each original risk category was used as a comparison metric for the follow-up validation. The diabetes was diagnosed using WHO criteria (2006).

## Results

### Development of Model

#### 1. Characteristics of Baseline Data

The total number of this database was 16246 (1009 subjects with diabetes). Characteristics of 15237 subjects without diabetes on the baseline survey are shown in Table 1.

**Table 1:** Characteristics of model and follow-up subjects (Mean ± SD).

	2001 baseline data			2001 follow-up data			2007 follow-up data		
	Men	Women	Total	Men	Women	Total	Men	Women	Total
Number	8624	6613	15237	1217	1071	2288	1217	1071	2288
Age(years)	46.52±16.2	47.47±14.8	46.01±15.63	50.41±14.62	49.65±13.21	50.05±13.98	55.99±14.71	55.23±13.29	55.63±14.07
BMI(kg/m^2^)	24.19±3.60	23.31±4.1	23.69±3.85	24.15±3.59	23.43±3.81	23.81±3.71	24.90±4.39	23.90±3.63	24.43±4.08
Waist(cm)	84.87±9.50	76.57±9.80	80.81±10.32	85.21±8.96	77.56±8.74	81.63±9.65	89.31±9.18	83.59±9.68	86.64±9.84
SBP(mmHg)	121±16.0	115.6±18	117.88±16.77	121.41±15.38	115.77±16.97	118.77±16.38	125.67±16.13	121.03±16.97	123.50±16.68
DBP(mmHg)	78.8±10.20	74.58±10.30	76.73±10.39	78.42±9.22	74.24±9.42	76.46±9.54	79.64±9.44	75.93±9.13	77.90±9.48
GLU(mmol/L)	4.91±1.27	4.9±1.20	4.70±0.62	4.74±0.62	4.76±0.61	4.75±0.61	4.90±0.84	4.87±0.80	4.89±0.82
CHOL(mmol/L)	4.82±0.95	4.97±1.10	4.85±0.99	4.88±0.86	5.06±0.99	4.97±0.93	5.09±0.91	5.36±1.00	5.22±0.96
TG(mmol/L)	1.61±1.26	1.39±1.00	1.48±1.09	1.55±0.95	1.44±1.00	1.50±0.98	1.64±1.00	1.51±1.06	1.58±1.03
HDL(mmol/L)	1.26±0.30	1.47±0.34	1.36±0.33	1.27±0.31	1.46±0.34	1.36±0.34	1.19±0.28	1.39±0.31	1.29±0.31
PSH (%)	13.73	17.8	14.5	3	5	4	3	5	4

#### 2. Results of Variable Selection

Nine variables were selected as risk variables for the risk assessment model both by a C4.5 decision tree and by logistic regression. They were: AGE, SEX, BMI, WAIST, CHOL, TG, HDL, DBP and PSH.

The occurrence frequencies (number) of each variable in all decision trees are shown in Table S3 in File S1. The variables whose frequencies of occurrence were higher than four in the first eight levels were chosen as the most relevant variables.

The results of the multivariate logistic regression are shown in Table S4 in File S1, the variables whose P value was lower than 0.05 were chosen as the strong relevant variables.

Although the P value of HDL was higher than 0.05 in the multivariate logistic regression, HDL is a well-known protective factor for diabetes and this was strongly suggested by the decision trees. Meanwhile, the empirical experience showed that the CHOL had a high relevance to diabetes in women aged around 50. So HDL and a new variable obtained by multiplying the CHOL and sex were both selected as risk variables.

The value of glycemia around 5.85 was used as the absolute criteria of pre-diabetes in the risk assessment model.

#### 3. Results of the Fast Clustering

The diabetes-related metabolic characteristics were well represented in the three groups by fast clustering using nine selected variables (Table S5 in File S1). The first and the biggest group represented the generally metabolic status of the adults. The second group indicated an alteration by aging and the third group was mainly composed of the persons with relatively high value of risk variables. The feature extracted by fast clustering offered a base for further risk assessment for diabetes with these common metabolic variables.

#### 4. Results of Relative Risk Calculation

**Figure 2:**
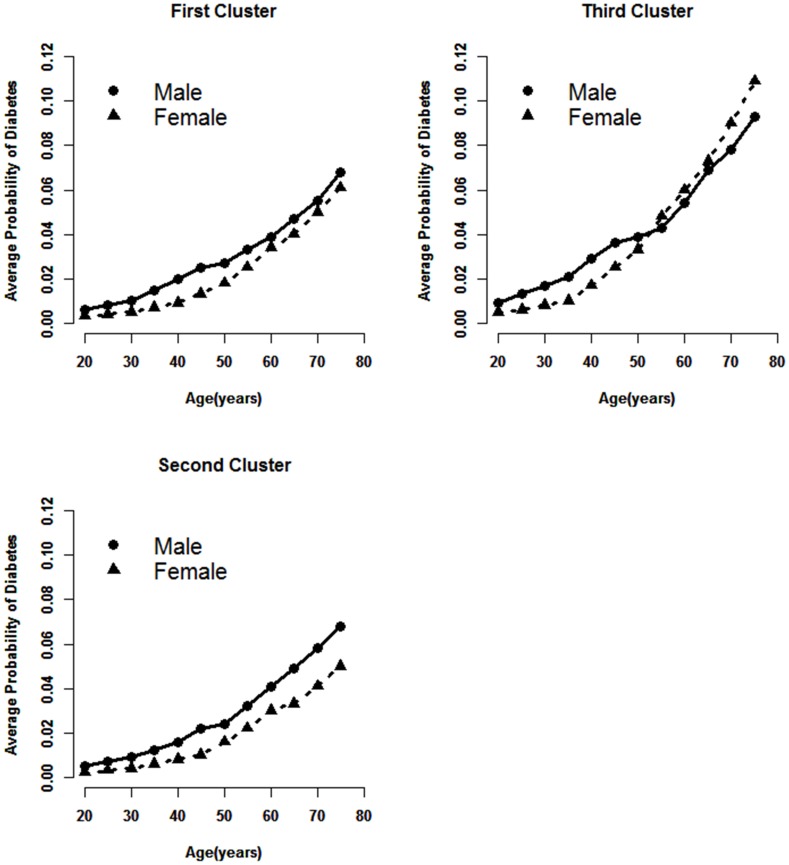
Average probability of diabetes in three clusters with each age and gender matching group.

The average probability by age and gender in the three clusters is shown in Figure 2.

As shown in Figure 2, the average probability of diabetes shows almost the same trend rising in parallel with age in the first and second cluster either for both men and women. An intersection of average probability between male and female presented at the age 55 in the third cluster. The average probability of diabetes was higher in women than in men. Furthermore, the average probability of diabetes in groups aged over 55 in the third cluster was higher than in the other clusters, especially in females.

#### 5. Comprehensive Risk Assessment

The procedure of the comprehensive risk assessment was implemented as in block A of Figure 1.

The model population (excluding persons with diabetes) was divided into four risk categories by this assessment model, the distribution of the degree of risk and the metabolic characteristics of each category are shown in Table 2.

**Table 2:** Distribution of risk degree and metabolic features of model population.

	Non Risk	Low Risk	Medium Risk	High Risk
Number	10186	2133	1639	1279
Percentage (%)	66.85	14	10.75	8.39
Age(years)	42.8±15.4	50.6±14.0	54.1±12.6	55.5±12.8
BMI(kg/m^2^)	22.3±2.6	26.4±4.1	26.9±4.1	27.8±4.6
Waist(cm)	76.9±8.4	88.1±8.8	89.7±8.1	92.8±10.3
GLU(mmol/L)	4.89±0.8	5.09±0.6	5.2±0.4	6.26±0.7
CHOL(mmol/L)	4.6±0.8	5.0±0.8	5.2±0.9	5.6±1.2
TG(mmol/L)	1.2±0.5	1.6±1.0	2.6±1.4	2.7±1.9
HDL(mmol/L)	1.4±0.3	1.3±0.2	1.2±0.2	1.2±0.3
SBP(mmHg)	112±13	119±17	124±15	128±17
DBP(mmHg)	73±8	82±9	82±8	87±9
PSH (%)	8.7	21.4	12.2	11.4

In this model population, around 20% of persons have a high and middle-high risk of diabetes. Meanwhile, the mean value of each variable increased significantly from the non-risk category to the high risk category except the HDL.

### Model Validation

#### 1. Cross-validation of Baseline Population

The accuracy of all risk determination by this risk assessment model was 90.99%, as shown in Table S6 in File S1.

#### 2. Follow-up validation

After six-year follow-up, the average incidence of diabetes in the model population was around 4.5 persons/1000 persons/year. The incidence increased significantly from the non-risk group (1.5 persons/1000 persons/year) to the high-risk group (28.2 persons/1000 persons/year). The incidence increased almost 19 times and 6 times in high-risk group than that in the non-risk and total group, respectively (Table 3). The characteristics of 2288 persons both in 2001 and 2007 are shown in Table 1.

**Table 3:** Incidence of diabetes of 6-years follow-up in 2288 subjects.

	Number of patients in different risk categories in 2001	Number of patients in different risk categories in 2007	Incidence of diabetes	
			Number of diabetes	per one thousand persons per year (95% CI)
Non Risk	1546	1165	14	1.5 (0.6–2.1)
Low Risk	313	457	9	4.8 (2.1–8.5)
Medium Risk	246	358	8	5.4 (1.7–8.8)
High Risk	183	246	31	28.2 (19.1–37.3)
Total	2288	2226	62	4.5 (3.3–5.7)

## Discussion

With nine common variables and one cut-off point value of glycemia as risk variables, a computerized assessment model was developed for the risk of type 2 diabetes in Chinese. The model was constructed by very common parameters including general personal information (age, gender, BMI, waist and blood pressure) metabolic variables (CHOL, TG, and HDL) and genetic factors (PSH), so this model is both available and affordable. The model could efficiently and accurately stratify the population into four different groups. The persons at high and medium risk will be targeted, and be thought of as persons at risk in the list of intervention.

The accuracy of the model was fully validated by the incidence of diabetes in the six-year follow-up data. The incidence of diabetes was dramatically increased from the non-risk persons (1.5 persons/1000 persons/year) to the high-risk persons (28.2 persons/1000 persons/year) during six-year follow-up of the model population. Furthermore, the percentage of high-risk persons could be clearly reduced by reassessment after six-month intervention (data was not shown).

Compared with different risk assessment tools that have been developed in the last ten years, the principle of thinking was quite similar. Including assessment variables, methods were proposed to account for risk, as well as certain criteria for determination status of risk. The main differences between our model and others are focused on two points: precision and efficiency. Taking the advantage of previous work [23], [24], [25], the core of this assessment was built on the comprehensive analysis from selected risk variables to calculate the probability of diabetes by age and gender, a way of assessment which is based on computer technology. This model could be used for risk determination without any limitation of numbers and give the results in several seconds either for screening or for reassessment. All these features will be greatly facilitate the practices of intervention for diabetes.

One of the problems in developing the assessment model was how to determine the status of diabetes risk not only derived from impaired fasting glucose but also from impaired glucose tolerance. However, the risk variables related to both impaired regulations of glucose metabolism were reported as being quite similar. But the sensitivity of these risk factors in the assessment of the two impaired regulations of glucose metabolism was quite different. The accuracy of assessment for impaired fasting glucose, such as in our model, reaches 90%. The accuracy, evaluated by the same model, of assessment for impaired glucose tolerance reduced to 68% (data not shown). This presents the big challenge of improving the assessment model to recognize the risk of impaired glucose tolerance.

In conclusion, a risk assessment model was developed for type 2 diabetes in Chinese. The model could stratify the population into four risk groups. The model could be used as a technique tool to support the screening of persons at risk of type 2 diabetes and to evaluate the integrating effects of intervention.

## Supporting Information

File S1 **Supplemental Material.** File S1 contains seven tables and one figure. They are: (1) Table S1 the optimal number of fastclus; (2) Table S2 Hosmer and Lemeshow test for three logistic regressions; (3) Table S3 frequency of selected variables occurrence in all decision trees; (4) Table S4 risk factors and beta coefficient derived from multivariate logistic regression; (5) S5 the characteristics of different clusters (mean±SD); (6) Table S6 the results of jackknife cross-validation in model population; (7) Table S7 the list of 96 variables in risk variable selection; (8) Figure S1 receiver operating characteristic curve of RR.
(DOC)
