# Supplementary material for: A Risk Assessment Model for Type 2 Diabetes in Chinese
Source: PLoS One. 2014 Aug 7;9(8):e104046. doi: 10.1371/journal.pone.0104046 (PMC4125170; doi:10.1371/journal.pone.0104046)

Supplemental Table S1 The optimal number of fastclus

| Number of cluster | R^2^ | R^2^_i+1_ – R^2^_i_(i=2,3,4) | CCC |
| --- | --- | --- | --- |
| 2 | 0.0683 |  | 325.8 |
| 3 | 0.12141 | 0.053 | 218.6 |
| 4 | 0.16478 | 0.0434 | 199.9 |
| 5 | 0.20138 | 0.0367 | 237.5 |

Supplemental Table S2 Hosmer and Lemeshow test for three logistic regressions

|  | Chi-square | Sig. |
| --- | --- | --- |
| First Cluster | 6.535 | 0.587 |
| Second Cluster | 5.881 | 0.661 |
| Third Cluster | 3.177 | 0.923 |

Supplemental Table S3 Frequency of selected variables occurrence in all decision trees

| Variables | GLU | AGE | SEX | | HDL | DBP | | P HIS | | WAIST | BMI | WEIGHT | | CHOL | TG |
| --- | --- | --- | --- | --- | --- | --- | --- | --- | --- | --- | --- | --- | --- | --- | --- |
| No. | 36 | 18 | 16 | | 13 | 13 | | 12 | | 9 | 8 | 7 | | 7 | 5 |
| GLU | GLU=7 | GLU=5.85 | | GLU=5.5 | | | GLU=5.25 | | GLU=4.06 | | | |  | | |
| No. | 9 | 6 | | 4 | | | 3 | | 3 | | | |  | | |

Supplemental Table S4 Risk factors and beta coefficient derived from multivariate logistic regression

| Variables | Beta | P value |
| --- | --- | --- |
| AGE | 0.027 | 0.000 |
| SEX | 0.369 | 0.096 |
| BMI | 0.028 | 0.098 |
| WAIST | 0.039 | 0.004 |
| CHOL | 0.224 | 0.170 |
| TG | 0.018 | 0.027 |
| HDL | 0.302 | 0.003 |
| DBP | 0.019 | 0.044 |
| PSH | 0.090 | 0.084 |
| Constant | -8.856 | 0.000 |

Supplemental Table S5 The characteristics of different clusters (mean±SD)

|  | First Cluster | Second Cluster | Third Cluster |
| --- | --- | --- | --- |
| Number | 9822 | 5885 | 539 |
| AGE(years) | 40.19±14.09 | 57.69±12 | 49.54±12.82 |
| BMI(kg/m^2^) | 22.42±2.92 | 25.90±4.31 | 26.13±3.43 |
| WAIST(cm) | 76.95±8.08 | 87.75±9.22 | 88.79±8.85 |
| CHOL(mmol/L) | 4.65±0.93 | 5.21±0.94 | 5.48±1.48 |
| TG(mmol/L) | 1.18±1.17 | 1.72±0.75 | 5.45±2.89 |
| HDL(mmol/L) | 1.43±0.33 | 1.26±0.28 | 0.96±0.23 |
| DBP(mmHg) | 72.16±7.87 | 84.65±9.43 | 80.92±9.47 |
| SEX(% of male) | 50.3 | 76.6 | 65.9 |
| PSH(%) | 15.9 | 14.1 | 14.8 |

Supplemental Table S6 The results of jackknife cross-validation in model population

|  |  | Assessment Value | | |
| --- | --- | --- | --- | --- |
|  |  | Non Risk | different degree of Risk | Diabetes |
| True Value | Non Risk | 74.98% | 25.03% | 0 |
|  | different degree of Risk | 9.01% | 90.99% | 0 |
|  | Diabetes | 0.31% | 22.53% | 77.16% |

Supplemental Table S7 The list of 96 variables in variable selection

| No. of variables | No. of selected variables | Abbreviation | Designation |
| --- | --- | --- | --- |
| 1 | 1 | AGE | Age |
| 2 |  | ALB | Albumin |
| 3 |  | ALKACID | Alkalinity acidity |
| 4 |  | AMYL | Amylaceum |
| 5 |  | ARRH | Arrhythmia |
| 6 |  | BYC | Whether do riding or not |
| 7 | 2 | CBVA | History of Cerebral Vascular Disease |
| 8 |  | CBVB | Whether take intervention for Cerebral Vascular Disease |
| 9 | 3 | CBVC | How long have you been CBV |
| 10 | 4 | CBVD | Parental history of Cerebral Vascular Disease |
| 11 | 5 | CBVSYM | Symptoms of Cerebral Vascular Disease |
| 12 |  | CEPH | Cephalalgia |
| 13 |  | CERBI | Cerebral infarction |
| 14 |  | CERPLOY | Cervical polyp |
| 15 |  | CERVSPON | Cervical spondylopathy |
| 16 | 6 | CHD | Coronary heart disease |
| 17 | 7 | CHOL | Cholesterol |
| 18 |  | CHTG | Chest tightness |
| 19 |  | CK | Creatine kinase |
| 20 |  | CR | Crea |
| 21 | 8 | DBP | Diastolic blood pressure |
| 22 |  | DEHY | Dehydrogenase |
| 23 | 9 | DIETA | Dietary habit |
| 24 | 10 | DIETB | Daily of salt intake |
| 25 | 11 | DIETC | Daily of fat intake |
| 26 | 12 | DIETD | Daily of staple food intake |
| 27 | 13 | DIZZ | Whether feel dizziness or not |
| 28 | 14 | DMA | History of Diabetes |
| 29 | 15 | DMB | Whether take intervention for Diabetes |
| 30 | 16 | DMC | How long have you been diabetes |
| 31 | 17 | DME | Parental history of diabetes |
| 32 | 18 | DRINK | History of Drinking |
| 33 | 19 | EDU | Educational Background |
| 34 | 20 | ETHNIC | Ethnics |
| 35 | 21 | EXERB | Quality of exercise |
| 36 | 22 | EXERC | Whether do exercise or not |
| 37 | 23 | EXERD | Frequency of exercise |
| 38 |  | Fe | Serum Iron |
| 39 | 24 | GLU | Fasting plasma glucose |
| 40 |  | GPT | Glutamic-Pyruvic Transaminase |
| 41 |  | GT | Transpeptidase |
| 42 |  | HBA1C | HBA1C |
| 43 | 25 | HDL | High density lipoprotein |
| 44 | 26 | HEIGHT | Height |
| 45 |  | HGASTR | History of gastropathy |
| 46 | 27 | HIP | Hip circumference |
| 47 | 28 | HPA | History of Hypertension |
| 48 |  | HPB | Whether take intervention for Hypertension |
| 49 | 29 | HPD | Parental history of Hypertension |
| 50 | 30 | HRTA | History of heart disease |
| 51 |  | HRTB | Whether take intervention for heart disease |
| 52 | 31 | HRTD | Parental history of heart disease |
| 53 | 32 | HRTSYM | Symptoms of heart disease |
| 54 |  | HYDRX | Hydroxybutyrate |
| 55 | 33 | HYPLIPA | History of hyperlipidemia |
| 56 |  | HYPLIPAB | Whether take intervention for hyperlipidemia |
| 57 | 34 | HYPLIPAD | Parental history of hyperlipidemia |
| 58 | 35 | JOB | Job |
| 59 | 36 | JOBA | Nature of work |
| 60 | 37 | JOBC | How many days a week for working |
| 61 | 38 | JOBD | How long do working per day |
| 62 | 39 | JOBSTR | Working pressure |
| 63 |  | KETOB | Ketobodies |
| 64 | 40 | KIDN | History of kidney disease |
| 65 |  | KIDNB | Whether take intervention for kidney disease |
| 66 | 41 | KIDND | Parental history of kidney disease |
| 67 | 42 | LDL | Low density lipoprotein |
| 68 | 43 | LIVER | History of liver disease |
| 69 | 44 | LUMB | Lumbar disease |
| 70 | 45 | MARRIAGE | Marriage status |
| 71 |  | OB | Occult blood |
| 72 |  | POT | Potassium |
| 73 |  | PROT | Protein |
| 74 | 46 | PSYSTR | Psychological stress |
| 75 |  | RA | Arthritis |
| 76 |  | RBC | Red blood cell |
| 77 | 47 | SBP | Systolic blood pressure |
| 78 | 48 | SEX | Gender |
| 79 | 49 | SLPA | Sleep |
| 80 | 50 | SLPB | Sleep quality |
| 81 | 51 | SMK | History of smoking |
| 82 |  | SMKA | Whether stop smoking or not |
| 83 |  | SS | Sjogren syndrome |
| 84 |  | TBIL | Total bilirubin |
| 85 | 52 | TC | Total cholesterol |
| 86 | 53 | TG | Triglyceride |
| 87 |  | THRL | Thorcalgia |
| 88 |  | THYREO | Thyreoitis |
| 89 | 54 | TUMOUR | Tumour |
| 90 |  | UA | Uric acid |
| 91 |  | UN | Urea Nitrogen |
| 92 |  | URO | Urobilinogen |
| 93 | 55 | VLDL | Very low density lipoprotein |
| 94 | 56 | WAIST | waist |
| 95 |  | WBC | white blood cell |
| 96 | 57 | WEIGHT | Weight |


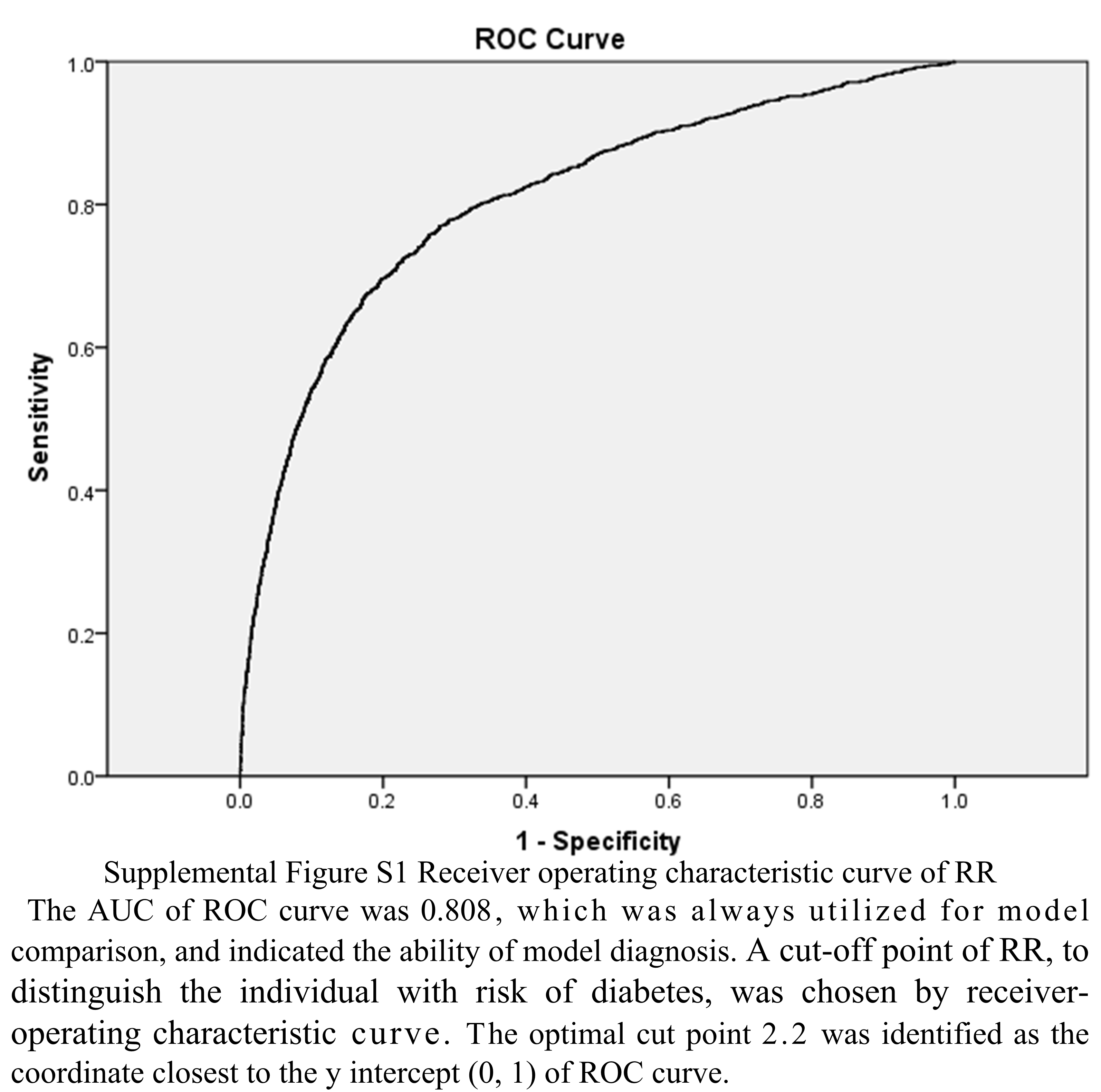

Supplement: File S1 — Supplemental Material. File S1 contains seven tables and one figure. They are: (1) Table S1 the optimal number of fastclus; (2) Table S2 Hosmer and Lemeshow test for three logistic regressions; (3) Table S3 frequency of selected variables occurrence in all decision trees; (4) Table S4 risk factors and beta coefficient derived from multivariate logistic regression; (5) S5 the characteristics of different clusters (mean±SD); (6) Table S6 the results of jackknife cross-validation in model population; (7) Table S7 the list of 96 variables in risk variable selection; (8) Figure S1 receiver operating characteristic curve of RR. (DOC) [file pone.0104046.s001.doc]
